# Nodular subcutaneous infiltrates in a kidney transplant recipient: lessons from a case

**DOI:** 10.1007/s40620-022-01354-5

**Published:** 2022-05-26

**Authors:** Michael Kolland, Sabine Zitta, Eva-Maria Hassler, Lisa Kriegl, Ines Zollner-Schwetz, Alexander R. Rosenkranz, Alexander H. Kirsch

**Affiliations:** 1 Division of Nephrology, Medical University of Graz, Auenbruggerplatz 27, 8036 Graz, Austria; 2 Division Neuroradiology, Vascular, and Interventional Radiology, Department of Radiology, Medical University of Graz, Graz, Austria; 3 Division of Infectious Diseases, Department of Internal Medicine, Medical University of Graz, Graz, Austria

**Keywords:** Kidney transplantation, Immunosuppression, Infection

## Case presentation

**Fig. 1:**
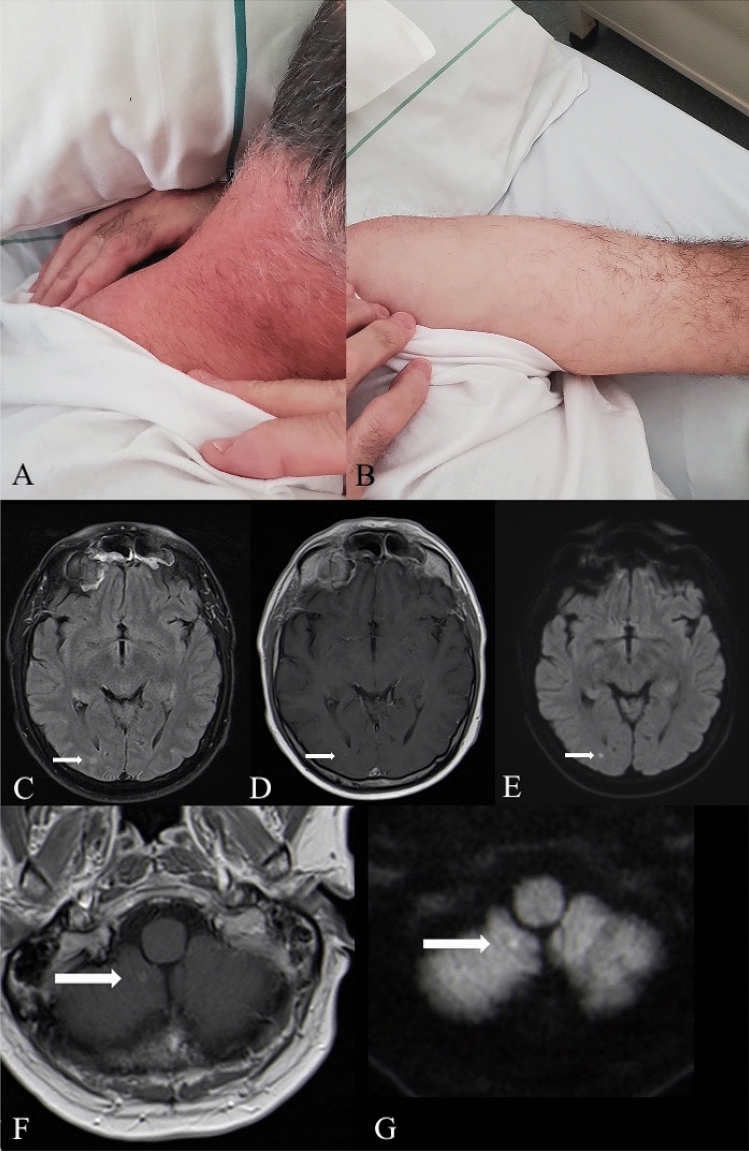
Cutaneous and intracranial lesions (**A**–**G**). **A** shows cutaneous involvement with nodular bulging of the nuchal region (**A**) and (**B**) upper left arm. **C**–**E** shows images of the performed MRI examination of the neurocranium on a 1.5 Tesla scanner. (MAGNETOM Solar, Siemens, Erlangen, Germany). The panel shows hyperintense signal in the TIRM sequence (**C**), annular contrast enhancement in contrast enhanced T1-weighted sequences (**D**) and diffusion restriction in diffusion weighted sequence (**E**). A small cerebellar lesion is shown in the posterior cerebellar lobe of the right hemisphere in contrast enhanced (**F**) and diffusion weighted (**G**) images

A 52-year-old man, scheduled for a routine visit, presented with nodular infiltrates, measuring 2–4 cm in diameter, located at the neck, and the upper right arm (Fig. 1A, B), which had appeared a week prior to presentation, initially non-tender, then painful. No history of fever, shivers, previous injuries, or sweating was reported. He worked as a farmer and had close contact with livestock, including chickens, a considerable number of which had died during the previous weeks.

His past medical history was notable for renal transplantation three years prior to presentation. The underlying renal disease was relapsing PR3-ANCA-associated vasculitis with renal, pulmonary and ocular involvement, which had initially been treated with rituximab followed by azathioprine maintenance therapy. At the time of presentation, he was on stable maintenance immunosuppression with tacrolimus and prednisolone and prophylaxis with valganciclovir and sulfametrol 800 mg/trimethoprim 160 mg half a dose 3 times a week. His baseline kidney function prior to admission averaged eGFR of 40 ml/min/1.73 m^2^, therefore KDIGO stage CKD G3b.

On physical exam, the reddened lesions were partly coarse, partly liquid appearing (Fig. 1A, B). Sonography of the lesion showed changes consistent with abscess formation for which he was empirically started on oral clindamycin. On day three, he returned to the outpatient clinic with newly formed lesions on the upper left arm and right leg accompanied by acute deterioration overnight with night sweats, severe headache, cough, and fever. His lab work was remarkable for mild leukocytosis (12.9 × 10^9^/mL), neutrophilia (80%), elevated C-reactive protein (CRP) (90 mg/L), elevated erythrocyte sedimentation rate (33 mm/hr.), and acute kidney injury (AKI I creatinine from baseline 1.5–2.1 mg/dL). A SARS-CoV-2 PCR returned positive and the patient was admitted to the inpatient ward.

Blood cultures were drawn and drainage and cultures of the abscess, as well as an immunologic serological profile were performed on the day of admission. Empirical antibiotic therapy was adapted to a broader spectrum with ceftriaxone and doxycycline. Magnetic resonance imaging (MRI) of the skull and cervical spine and CT-scan of the chest were ordered on days 4 and 5, respectively .

Blood cultures and the immunology returned negative. MRI revealed small nodular occipital and cerebellar lesions (Fig. 1C–G) and CT-scan showed patchy ground-glass-like lesions (Fig. 1C–G).

Abscess drainage grew Gram-positive rods. Cultures from abscess drainage revealed *Nocardia farcinica* using 16S rRNA gene sequencing and MALDI-TOF MS. Diagnosis of disseminated cutaneous Nocardiosis with cerebral and suspected pulmonary involvement was established. The cranial lesions corresponded to abscesses in the context of nocardiosis, see Fig. 1C–G, and CT scan was consistent with both COVID-19 pneumonia, as well as pulmonary nocardiosis. Due to the broad sensitivity pattern, ceftriaxone 2 g twice daily and doxycycline 200 mg once daily were initially maintained, but because of the cerebral lesions and increasing CRP on day 7, anti-infectives were changed to linezolid 3 × 600 mg, meropenem 3 × 2 g and doxycycline (minimal inhibitory concentration (MIC) 8 µg/mL). Consequently, skin lesions, oxygen requirement and CRP (8 mg/L) considerably decreased, but clinical deterioration recurred on day 17, necessitating adjustments to amikacin (MIC 1 µg/mL) plus ceftriaxone (MIC 4 µg/mL) and tedizolid. The patient’s respiratory affliction further worsened to acute respiratory distress syndrome (ARDS). He was subsequently transferred to the intensive care unit on day 18. Unfortunately, clinical stabilization could no longer be achieved, and the patient died of multiorgan failure 3 weeks after admission.

## Lessons for the clinical nephrologist

Following solid organ transplantation, patients are prone to infections due to multiple reasons, such as immunosuppression, surgical and environmental factors [1].

While surgical complications are the leading causes of infection during the first month after transplantation, opportunistic infections most often occur from month two to six, and after six months transplantation recipients commonly suffer the same infections as the general population.

Influenza, urinary tract infections and pneumococcal pneumonia are frequently observed, but also varicella zoster virus (VZV) reactivation or cytomegalovirus (CMV) viremia may occur. Furthermore, patients at greater immunological risk, such as those with episodes of acute or chronic rejection who require higher levels of immunosuppression are especially at risk. Typical opportunistic agents include *C. neoformans, P. jirovecii, L. monocytogenes* and *Nocardia *spp. [1] Nocardial infections are an infrequent cause of infection with a yearly incidence of 0.39–0.55/100,000 [2] and typically occur in kidney and heart transplant recipients. One-year mortality is tenfold higher in solid-organ transplant recipients with nocardiosis compared to recipients without nocardiosis (16.2% versus 1.3%) [3], thus, early diagnosis and adequate therapy is quintessential. Outcomes in solid-organ transplant recipients are reported in supplemental Table 1. Treatment includes trimethoprim/sulfamethoxazole, which is also part of pneumocystis prophylaxis in patients with underlying pulmonary disease receiving a solid organ transplant.

Whether direct transmission from the patient's chickens was the causative factor remains unclear, but outbreaks in livestock and indeed in poultry farms have been described. Case reports of people with Nocardia and chicken exposure appear in the literature as well but due to the tragic course of events, we were unable to pursue this association. Certainly, for people raising livestock, unavoidable contact with soil, organic material and water are a potential source of infection.

*N. farcinica* is amongst the most common species of Nocardia in central Europe with increasing evidence in rodents which suggests that *Nocardia farcinica* is more virulent compared to other Nocardia species [4]. Therefore, it is crucial to obtain resistance testing early to enable targeted antimicrobial therapy. In our case, tissue sampling upon first presentation may have altered therapy and outcome.

Clinically, there are multiple possible manifestations, including skin and pulmonary involvement, while the most common route of entry is via pulmonary infection/inhalation of dust [5].

Patients with defects in T-cell mediated immunity, i.e. patients after solid organ transplantation, are susceptible to this pathogen. Risk factors of nocardiosis coincided in this patient: corticosteroid therapy and the use of calcineurin inhibitors (tacrolimus).

Frequently, patients present with productive or nonproductive cough, shortness of breath, fever, night sweats and progressive fatigue due to pulmonary nocardiosis. CT scans of the chest may show nodules, cavitations, diffuse alveolar pulmonary infiltrates, lung abscesses, or pleural effusions [6, 7].

Cutaneous disease is commonly preceded by a local injury to the skin, which may disseminate in immunocompromised patients. Differential diagnosis of cutaneous abscesses should include staphylococcal infection, as the most common cause, however other bacterial, fungal, or mycobacterial infections must also be considered in any immunocompromised patient.

Clinically, this manifestation is not distinguishable from other bacterial pathogens as they resemble each other, i.e. small, staphylococcal-like lesions (making early comprehensive microbiological diagnosis indispensable).

Cultivation of *Nocardia* can be difficult, since it is a slow-growing pathogen. Within samples of mixed flora, however, the *Nocardia* colonies are readily obscured by colonies of faster-growing bacteria, while decontamination methods used for mycobacterial cultures, for example, are too harsh for Nocardia species and can significantly limit sample viability [8]. Treatment depends not only on the clinical manifestations (cutaneous only versus disseminated) but also on the susceptibility pattern of clinical isolates which can vary considerably. Initial treatment in disseminated disease should consist of 2–3 agents, and may include trimethoprim/sulfamethoxazole, amikacin, ceftriaxone, meropenem, linezolid and doxycycline [7]. The optimal treatment duration has not been determined, but a prolonged course of 3–6 months for cutaneous and 6–12 months for disseminated disease is usually necessary.

In our case, initial antibiotic therapy was adapted according to the antibiogram. The lack of improvement on initial therapy with clindamycin can partly be explained by the high MIC of > 256 µg/ml found later. Patient’s prophylaxis with sulfamethoxazole/trimethoprim showed high MIC (> 32 µg/ml) values. Unfortunately, no clear EUCAST breakpoints have been defined to date. A recent retrospective study suggested that low dose co-trimoxazole does not prevent nocardial infections [9].

This case illustrates the importance of keeping in mind a broad differential of potential pathogens in immunosuppressed hosts with infections. We acknowledge that the source of infection (most likely infected poultry) has not definitively been shown.

Disseminated nocardiosis must be considered as a differential diagnosis in transplant recipients, since it mainly occurs in immunocompromised patients, and may be fatal when disseminated, therefore, early diagnosis is crucial.

## Supplementary Information

Below is the link to the electronic supplementary material.

Supplementary file1 (DOCX 21 kb)

## Data Availability

Data sharing is not applicable to this article as no datasets were generated or analyzed during the current study.

## Abbreviations

AKI: Acute kidney injury
ARDS: Acute respiratory distress syndrome
Covid-19: Corona virus disease-19
CRP: C-reactive protein
CT: Computer tomography
DWI: Diffusion weighted imaging
EUCAST: European Committee on Antimicrobial Susceptibility Testing
MIC: Minimum inhibitory concentration
MRI: Magnetic resonance imaging
PR3-ANCA: Proteinase-3-Anti-Neutrophil-Cytoplasmatic-Antibodies
SARS-CoV-2: Severe acute respiratory syndrome coronavirus type 2
SCr: Serum Creatinine
TIRM: Turbo inversion recovery magnitude
VZV: Varicella-zoster virus

## References

[1] Patel R, Paya CV (1997). Infections in solid-organ transplant recipients. Clin Microbiol Rev.

[2] Minero MV, Marin M, Cercenado E, Rabadan PM, Bouza E, Munoz P (2009). Nocardiosis at the turn of the century. Med (Baltim).

[3] Lebeaux D, Freund R, van Delden C, Guillot H, Marbus SD, Matignon M (2017). Outcome and treatment of nocardiosis after solid organ transplantation: new insights from a european study. Clin Infect Dis.

[4] Desmond EP, Flores M (1993). Mouse pathogenicity studies of Nocardia asteroides complex species and clinical correlation with human isolates. FEMS Microbiol Lett.

[5] Wilson JW (2012). Nocardiosis: updates and clinical overview. Mayo Clin Proc.

[6] Kim J, Kang M, Kim J, Jung S, Park J, Lee D (2016). A Case of Nocardia farcinica Pneumonia and Mediastinitis in an Immunocompetent Patient. Tuberc Respir Dis (Seoul).

[7] Nocardiosis [Internet]. StatPearls Publishing. 2020 [cited January 23rd 2021]. Available from: https://www.ncbi.nlm.nih.gov/books/NBK526075/.

[8] Ashdown LR (1990). An improved screening technique for isolation of Nocardia species from sputum specimens. Pathology.

[9] Coussement J, Lebeaux D, van Delden C, Guillot H, Freund R, Marbus S (2016). Nocardia infection in solid organ transplant recipients: a multicenter European case-control study. Clin Infect Dis.

