# Supplementary material for: Nodular subcutaneous infiltrates in a kidney transplant recipient: lessons from a case
Source: J Nephrol. 2022 May 26;35(7):1919–22. doi: 10.1007/s40620-022-01354-5 (PMC9133818; doi:10.1007/s40620-022-01354-5)
Supplement: Supplementary file 1 — Supplementary file1 (DOCX 21 kb) [file 40620_2022_1354_MOESM1_ESM.docx]

Supplemental table 1: outcome of nocardiosis in solid-organ transplant recipients

| **First author** | **Journal** | **Year of publication** | **Country of publication** | **Included SOT patients** | **Outcome** |
| --- | --- | --- | --- | --- | --- |
| Harris et al.(1) | Mayo Clinic Proceedings | 2021 | USA | 54 | 25% Mortality at 6 months |
| Majeed et al.(2) | Transplant Infectious Diseases | 2018 | USA | 54 | 11% at 1 year |
| Lebeaux et al.(3) | Clin Infectious Diseases | 2017 | Europe | 117 | 16% at 1 year |
| Peleg et al.(4) | Clinical Infectious Disease | 2007 | USA | 35 | 14% at 6 months |

Table 1. SOT solid-organ transplant

1. Harris DM, Dumitrascu AG, Chirila RM, Omer M, Stancampiano FF, Hata DJ, et al. Invasive Nocardiosis in Transplant and Nontransplant Patients: 20-Year Experience in a Tertiary Care Center. Mayo Clin Proc Innov Qual Outcomes. 2021;5(2):298-307.

2. Majeed A, Beatty N, Iftikhar A, Mushtaq A, Fisher J, Gaynor P, et al. A 20-year experience with nocardiosis in solid organ transplant (SOT) recipients in the Southwestern United States: A single-center study. Transpl Infect Dis. 2018;20(4):e12904.

3. Lebeaux D, Freund R, van Delden C, Guillot H, Marbus SD, Matignon M, et al. Outcome and Treatment of Nocardiosis After Solid Organ Transplantation: New Insights From a European Study. Clin Infect Dis. 2017;64(10):1396-405.

4. Peleg AY, Husain S, Qureshi ZA, Silveira FP, Sarumi M, Shutt KA, et al. Risk factors, clinical characteristics, and outcome of Nocardia infection in organ transplant recipients: a matched case-control study. Clin Infect Dis. 2007;44(10):1307-14.
